# Laser Processing of Ti Contacts for Ohmic Behavior on P‑Type 4H-SiC

**DOI:** 10.1021/acsaelm.5c01338

**Published:** 2025-09-16

**Authors:** Roberto Vabres, Gabriele Bellocchi, Corrado Bongiorno, Marilena Vivona, Fabrizio Roccaforte, Paolo Badalà, Paola Mancuso, Valeria Puglisi, Simone Rascunà, Isodiana Crupi

**Affiliations:** † Engineering Department, 18998 University of Palermo, Palermo 90128, Italy; ‡ STMicroelectronics, Catania 95121, Italy; § CNR-IMM, Catania 95121, Italy

**Keywords:** silicon carbide, ohmic contact, pulsed laser annealing, phase composition, merged PiN Schoktty

## Abstract

This work explores a key challenge in power device fabrication: the formation of ohmic contacts on p-type 4H-silicon carbide (SiC). We demonstrate a selective, low thermal budget approach using single titanium (Ti) metallization combined with pulsed laser annealing (PLA), as an alternative to both metallic multilayer stacks and conventional high-temperature annealing. By applying PLA with fluences above 3.6 J/cm^2^, Ti contacts exhibit linear current–voltage (*I*–*V*) behavior, indicating effective ohmic contact formation, with over 50% improvement in conduction observed at 3.8 J/cm^2^. Cross-sectional transmission electron microscopy (TEM) and elemental mapping reveal that higher fluences promote deeper SiC consumption, and the formation of a continuous, epitaxially regrown SiC layer, bonded to a uniform titanium carbide (TiC) layer extended deeper into the p-doped region. This structure supports efficient charge transfer and strong interfacial bonding. Furthermore, increasing fluence drives the transient liquid phase composition from Ti-rich toward a more balanced Ti–Si–C composition, promoting the formation of ternary phases enriched in Si and C that enhance interfacial stability and electrical performance. This work demonstrates that PLA offers precise control over interfacial reactions and contact microstructures, offering a scalable, selective, and thermally efficient approach for ohmic contacts on p-type 4H-SiC, advancing the development of high-performance, next-generation SiC-based power electronics.

## Graphical abstract

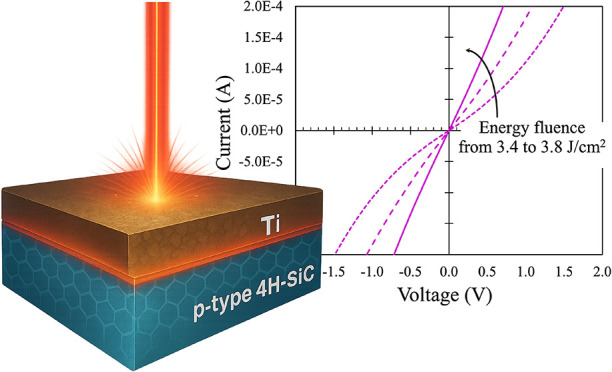

## 1 Introduction

Following a path like the one silicon (Si) set more than 30 years ago, the semiconductor technology landscape is undergoing significant evolution. Central to this progress is silicon carbide (SiC), a third-generation wide bandgap semiconductor material that has attracted considerable attention due to its superior electrical, thermal, and mechanical properties. These unique advantages position SiC at the forefront of next-generation semiconductor applications.^1–7^ Of the more than 250 SiC polytypes, 4H-SiC is the most widely utilized due to its combination of high electron mobility, wide bandgap, excellent thermal conductivity, and superior Baliga’s figure of merit.^8^ These properties make 4H-SiC a critical material for a broad spectrum of advanced technologies. For instance, it is extensively employed for the detection of ultraviolet (UV) light, emitted from natural and artificial sources. These UV photodetectors are used in environmental monitoring, space exploration, medical treatments, flame detection, and missile guidance systems.^9–15^ Additionally, 4H-SiC is particularly suitable for high-power, high-frequency, and high-temperature electronic applications.^16–24^ It is a key material for the development of efficient power converters, electric vehicles, renewable energy systems, and industrial electronics.^25–33^ In these applications, 4H-SiC’s unique properties enable a more efficient power conversion, reduce cooling requirements, and significantly enhance the longevity and reliability of devices. Moreover, the compatibility of SiC with conventional Si processing techniques has facilitated its widespread adoption, integrating it into existing semiconductor manufacturing infrastructures and accelerating its commercialization and widespread adoption.^34^ As a result, SiC is a cornerstone of modern power electronics and high-performance devices.

Despite the considerable progress in advancing SiC material and device technology since the 1980s^35,36^ several challenges still remain to be addressed.^37–41^ One of the most critical ones is the formation of reliable ohmic contact, especially on p-type SiC.^42–48^ Indeed, compared to Si, forming ohmic contacts on SiC is significantly more difficult, primarily due to its wide bandgap, which leads to higher Schottky barrier heights. For n-type 4H-SiC, various metal combinations, mainly based on nickel (Ni), have been extensively studied.^49–51^ These contacts are formed by rapid thermal annealing (RTA) process, typically above 900 °C, which induces deep diffusion of Ni atoms into the SiC, and a solid-state reaction between Ni and Si atoms, resulting in the formation of various nickel silicide (Ni_*x*_Si_*y*_) phases, along with the out-diffusion of C atoms.^52^ However, during the fabrication process of SiC power devices, the RTA method poses challenges due to its thermal budget, as it exposes the entire structure rather than just the contact region to high temperatures. Laser Thermal Annealing (LTA) is emerging as a promising alternative for the fabrication of titanium (Ti) based ohmic contacts on n-type 4H-SiC,^53–56^ with simulations demonstrating that the irradiated contact surface can reach temperatures up to 2000 °C while maintaining the opposite face of the device at a low temperature (*T* < 600 °C).^57^ However, forming ohmic contacts on p-type SiC remains a significant challenge due to the high Schottky barrier and the low acceptor ionization at room temperature. To date, most approaches have focused on metallization stacks, such as Ti/Al, Ni/Al, Pt- based etc.^58^ Shier et al.^59^ first proposed Cu–Ti and Al–Si eutectic alloys to form Ohmic contacts on p-type SiC, which, however, exhibited very high contact resistivity, ρ_c_. Over the past decades, extensive research has been devoted to developing Ohmic contacts for p-type SiC, with Ti/Al-based metal stacks delivering ρ_c_ values on the order of 10^–5^ Ω·cm^2^, and in some cases as low as 10^–6^ Ω·cm^2^ after high-temperature annealing above 1000 °C.^60,61^ To lower the annealing temperature to around 800 °C, additional metals such as Ni/Ti/Al, and Ge/Ti/Al have been incorporated into the metallization stack.^62–64^ Vivona et al.,^65^ for example, achieved a ρ_c_ of 5.8 × 10^–4^ Ω·cm^2^ for Ti/Al/W contacts on Al-implanted 4H-SiC after annealing at 1100 °C in argon. In contrast, we propose an innovative strategy by employing a single Ti metallization, not a Ti-based multilayer stack, combined with pulsed laser annealing (PLA). Unlike Ni, Ti does not tend to form silicide protrusions that can extend beyond the contact region impacting the device’s functionality. Unlike RTA, PLA delivers rapid, highly localized heating with precise energy control, significantly reducing the overall thermal budget of the process. PLA delivers ultrafast, highly vertical localized heating confined primarily to the metal/SiC interface, minimizing thermal exposure of adjacent device areas. This selective heating reduces risks of damage to temperature-sensitive layers, which are common concerns in conventional RTA.

This work aims to push the boundaries of contact engineering, providing valuable insights into the formation of Ti ohmic contacts on p-doped 4H-SiC, using nanosecond, nonequilibrium pulsed laser irradiation. While RTA exposes the entire substrate to high temperatures, PLA offers rapid, precisely controlled energy delivery directly to the Ti/SiC interface. This targeted approach allows for rapid thermal cycling with minimal impact on surrounding materials, making it particularly advantageous for advanced device architectures that require selective contact formation. The ultrafast heating and cooling rates of laser processing promote the formation of favorable crystalline interfacial phases, leading to improved contact performance, shorter processing times, and better compatibility with temperature-sensitive device layers. Realizing these benefits in practical applications requires a deeper understanding of the complex interfacial dynamics involved in the Ti/SiC reaction. While comprehensive assessments of surface morphology, stress evolution, and long-term reliability are beyond the scope of the present work, the results provide a solid foundation for future investigations. This study explores the influence of PLA parameters on the electrical and structural properties of Ti on p^+^ 4H-SiC contacts, providing insights for the optimization of contact engineering in next-generation SiC-based power devices. The method demonstrates highly efficient, localized contact formation with minimal thermal impact, making it well-suited for advanced device architectures. While challenges remain in adapting the process to smaller contact areas and densely packed layouts, these can be addressed by employing lasers with reduced spot sizes or advanced focusing optics to achieve precise, localized energy delivery, alongside selective masking techniques to confine the interaction region and protect sensitive areas from unintended exposure. With ongoing refinement, this approach holds strong potential for scalable integration into semiconductor manufacturing, paving the way for more compact, efficient, and robust SiC-based technologies.

## 2 Experimental Section

A commercially available n^+^ doped 4H-SiC wafer, with a resistivity of 20 mΩ·cm and a thickness of 350 μm, was used as the substrate for this study. The wafer was initially covered with an epitaxially grown n-type drift layer, with carrier concentration of 10^16^ cm^–3^ and a thickness of 5 μm, and, subsequently, selectively doped anode regions were formed using aluminum (Al) ion implantation, followed by thermal annealing at 1650 °C for 30 min in Argon atmosphere to activate dopants. The postannealing Al concentration profile, simulated using Synopsys tools, is shown in Figure S1 in the Supporting Information. To evaluate the ohmic behavior of single metal contacts on the p-type regions, rectangular Transmission Line Measurement (TLM) structures were fabricated. Each structure comprised 190 μm × 80 μm electrodes with spacings of 10, 30, and 50 μm. Metal contacts were formed by depositing either 100 nm of Ni or 20 nm of Ti. The structures were then subjected to RTA for 60 s, Ni at 1000 °C and Ti at 1100 °C in N_2_ atmosphere. The thinner Ti layer was chosen to enable efficient interface heating during laser processing, ensuring the reaction temperature for ohmic contact formation is reached without requiring excessive laser energy, thereby minimizing the impact on surrounding materials. The Ti layers were also processed using PLA, instead of the RTA step, with two pulses at wavelength of 310 nm, duration of 160 ns, and fluences of 3.4, 3.6, and 3.8 J/cm^2^, using a LT-3100 ns laser annealing system from SCREEN Semiconductor Solutions Co., Ltd. The two consecutive laser pulses were applied per spot with a 0.25 s delay between them. The laser spot size at the sample surface was approximately 1 cm^2^, and rastering was performed with no overlap.

*I*–*V* measurements were acquired with a two-terminal method at varying distances between the TLM pads on a manual Karl Suss Microtec probe station equipped with fine-tungsten tips using a HP 4156B parameter analyzer, with scanning source voltage swept from −2 to 2 V. Reproducibility was verified by repositioning the probes on several TLM pads located in different regions of the wafer, with only minimal variation observed. The microstructural characteristics, along with elemental mapping, were investigated using a 200 kV JEOL ARM200F Scanning Transmission Electron Microscope (STEM) equipped with a Gatan QuantumER Electron Energy Loss Spectroscopy (EELS) system.

## 3 Results and Discussion

Figure 1 shows the *I*–*V* characteristics acquired between adjacent pads of TLM structures, with electrode spacings of 10, 30, and 50 μm, for two different metal/p^+^ 4H-SiC structures after RTA treatments. As expected, due to the longer current path, the current decreases with increasing pad separation in both cases. For the 100 nm Ni sample annealed at 1000 °C, the *I*–*V* curves exhibit a clear linear behavior, indicating the formation of an effective ohmic interface. The corresponding total resistance values, *R*_T_, extracted from the slopes of the *I*–*V* curves, are shown in the inset as a function of the pad spacing, d. The high linearity of the data (correlation coefficient ≈ 1) enabled a reliable extraction of the sheet resistance, *R*_SH_, and ρ_c_ using the TLM model. Based on the linear fit, *R*_SH_ of 13471 ± 300 Ω/□ and ρ_c_ of (3.1 ± 0.9) × 10^–4^ Ω·cm^2^ were obtained. In contrast, 20 nm of Ti annealed at 1100 °C shows nonlinear *I*–*V* characteristics, possibly due to interfacial instability associated with the lack of a protective barrier^66^ and the limited film thickness, which prevents complete interfacial reaction.

**Figure 1:**
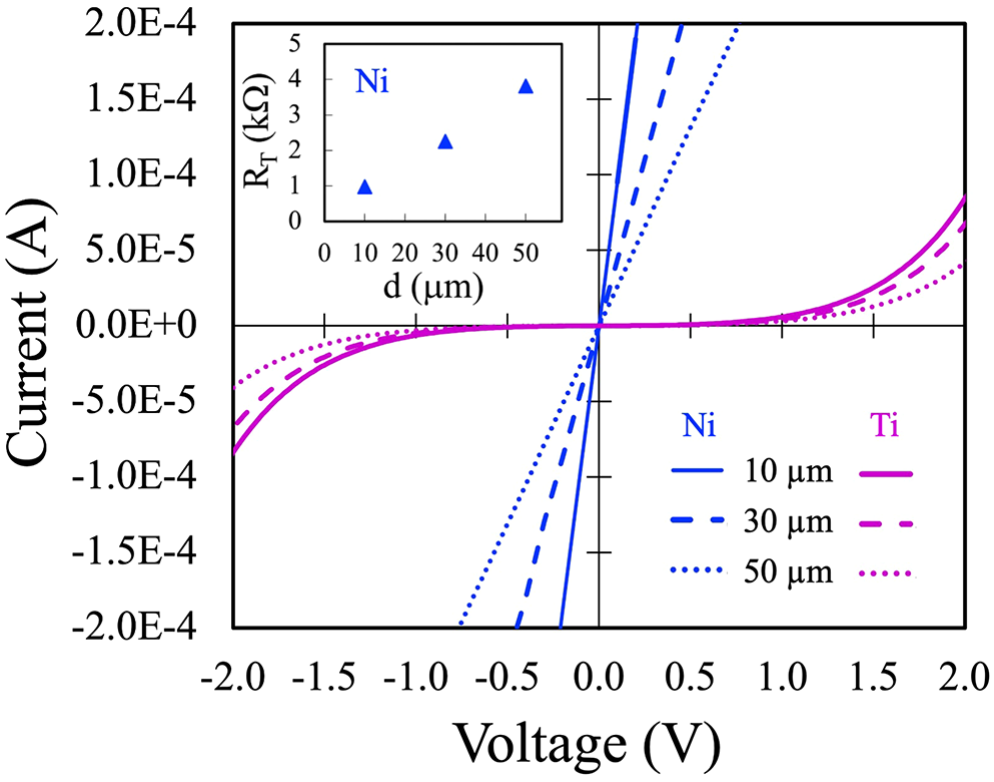
*I*–*V* characteristics, acquired between adjacent TLM pads, at three different distances of 10, 30, and 50 μm, fabricated by depositing on p^+^ 4H-SiC 100 nm of Ni and 20 nm of Ti followed by RTA at 1000 and 1100 °C, respectively. The inset shows R_T_ values for the 100 nm Ni sample annealed at 1000 °C.

Although Ni exhibits ohmic behavior in Figure 1, the high annealing temperatures required during RTA can lead to the formation of localized NiSi protrusions that can extend beyond the p^+^ region. These protrusions can lead to significant reliability issues in power devices, such as the Merged PiN Schottky (MPS) structure illustrated in Figure 2(a), prior to the deposition of the final continuous metal contacts on both the front and back sides. The whole MPS structure is carefully designed to combine the low forward voltage drop of a Schottky diode with the high blocking capability of a PN diode. This is achieved by embedding p^+^ anode regions into an n-type drift epilayer, which help suppress the electric field at the Schottky junction during reverse bias, improving breakdown voltage and reducing leakage. When an uncontrolled NiSi protrusion extends beyond the anode region and reach into the adjacent n-type drift layer, as shown by the circled area in the scanning electron microscopy (SEM) image in Figure 2(b), it can cause premature breakdown or leakage currents, due to the compromised junction isolation. To address this, Ti, which does not tend to form such invasive silicide protrusions, is here proposed in combination with PLA. Such technique offers precise, localized heating and helps preserve the structural and electrical integrity of the junction. For this reason, the thickness of Ti layer in Figure 1, which is thinner than the Ni one, was intentionally used in this study for comparison with PLA process. While this reduction in thickness is not critical under RTA, PLA benefits from a thinner metal layer, as it enhances energy absorption and promotes a more uniform heat distribution.

**Figure 2:**
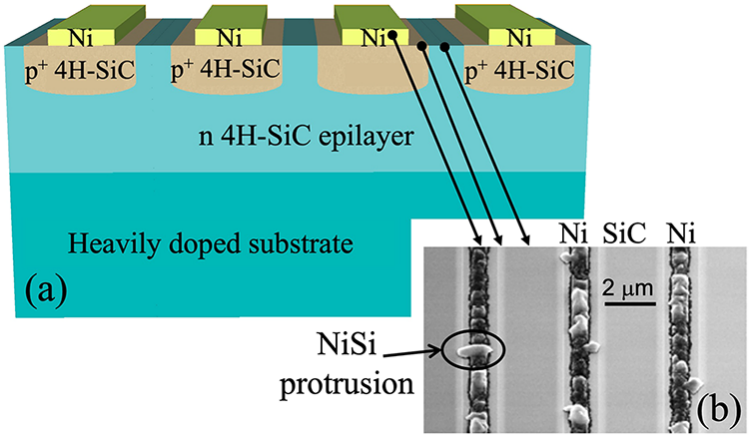
Schematic illustration (a) of Ni contacts on p-type implanted 4H-SiC in a MPS structure and the corresponding plan view scanning electron microscopy (SEM) (b) after RTA above 900 °C, revealing the formation of NiSi protrusion extending beyond the p^+^ 4H-SiC region.

The 20 nm of Ti deposited on p^+^ 4H-SiC was annealed using two laser pulses to provide highly localized heating and rapid cooling. The *I*–*V* characteristics in Figure 3, measured between adjacent contacts spaced 10 μm apart in the TLM structure, show a different behavior depending on the laser pulse fluences. When irradiated with 3.4 J/cm^2^, the *I*–*V* curves do not exhibit linearity, indicating insufficient formation of an ohmic contact. Vice versa, when the sample is irradiated with a fluence of 3.6 J/cm^2^, the curve is a straight line, and the conduction further increases at 3.8 J/cm^2^. For the samples irradiated at 3.6 and 3.8 J/cm^2^, the *R*_T_ values at three pads spacing are shown in the inset of Figure 3. Linear fits to these data reveal a slight decrease in *R*_SH_, from 13,733 ± 380 Ω/□ at 3.6 J/cm^2^ to 13,566 ± 277 Ω/□ at 3.8 J/cm^2^. More notably, ρ_c_ significantly decreases from (8.4 ± 0.29) × 10^–2^ to (2.9 ± 0.08) × 10^–2^ Ω·cm^2^, as the fluence increases, indicating improved contact quality. These ρ_c_ values, however, are higher than those measured for the 100 nm of Ni annealed under RTA at 1000 °C and are approximately two to 4 orders of magnitude greater than the best-reported Ti-based multilayer metallization systems. Nonetheless, the present results demonstrate the feasibility of forming Ohmic contacts on p-type SiC without multilayer stacks or high-temperature annealing, offering a simplified and potentially more compatible approach for certain device integration scenarios. When 20 nm of Ti on SiC are irradiated with two laser pulses at fluences above 2.6 J/cm^2^, temperature higher than 2000 K are expected at the contact surface,^53,67^ and a series of rapid interfacial reactions occur. These processes include the formation of a transient liquid phase which promotes rapid interdiffusion, atomic rearrangement and the development of localized crystalline features. After the irradiation, the Ti crystallizes and forms compounds with Si or C. These transformations significantly modify the microstructure at the metal/anode interface, thereby influencing its electrical performance.

**Figure 3:**
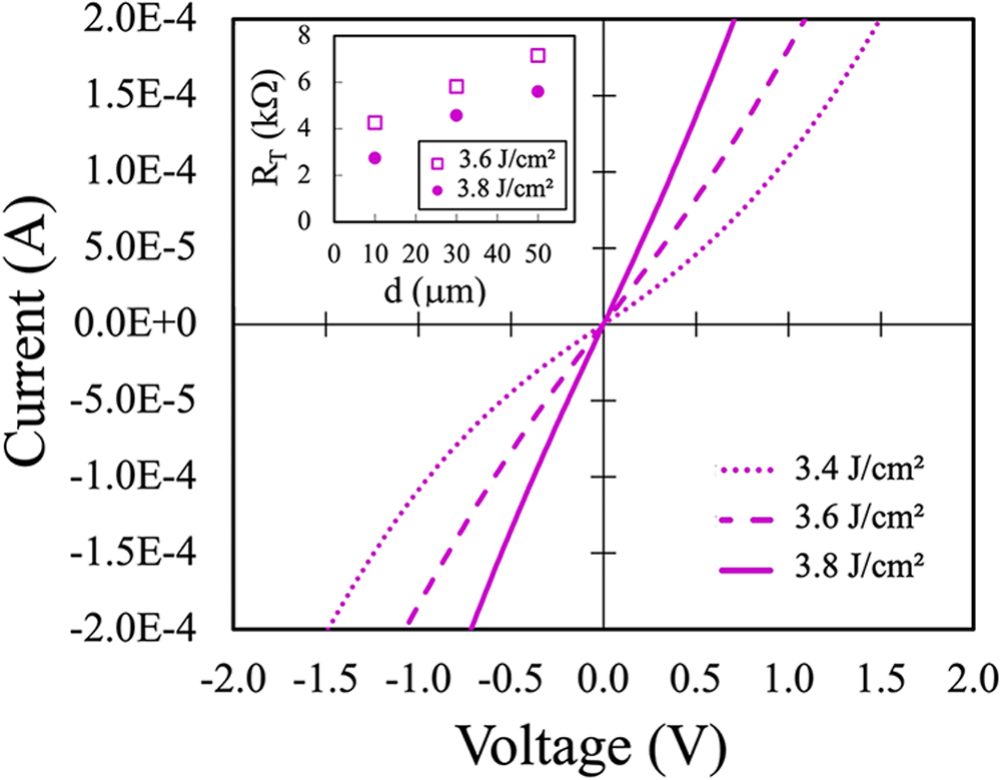
*I*–*V* characteristics, acquired between adjacent TLM pads, made with 20 nm of Ti, at distance of 10 μm, after two laser pulses at 3.4, 3.6, and 3.8 J/cm^2^ region. The inset shows *R*_T_ values for the samples irradiated at 3.6 and 3.8 J/cm^2^.

Indeed, the cross-sectional STEM images and the EELS maps in Figure 4 reveal distinct differences in interfacial morphology and elemental distribution between the two samples annealed with the lower and higher fluence conditions of Figure 3. For the sample irradiated at 3.4 J/cm^2^, Figure 4(a), limited reaction between Ti and SiC are observed. The Ti layer remains largely intact, the interface appears irregular and discontinuous, indicating limited interdiffusion and incomplete formation of interfacial reaction products. This is confirmed by the Ti map, Figure 4(b), which shows a strong and localized Ti signal within the deposited layer, and the Si map, Figure 4(c), where Si remains mostly confined to the SiC substrate, with only slight diffusion at the top of Ti layer. The C map, Figure 4(d), shows that carbon remains mostly within the SiC layer, although upward diffusion is evident. These results in the accumulation of carbon vacancies on the near surface of 4H-SiC and the formation of a TiC-based alloy layer that is not in direct contact with the underlying SiC region. Finally, the O map, Figure 4(e), shows a thin surface-localized SiO_2_ layer, likely from ambient exposure. In contrast, the sample irradiated at the maximum laser fluence exhibits a more continuous and uniform interfacial structure, Figure 4(f), indicating a stronger reaction between Ti and SiC. The Ti map, Figure 4(g), reveals a more diffuse distribution of Ti extending into the SiC region, while the Si map, Figure 4(h), and C map, Figure 4(i) indicate substantial intermixing of elements at the interface, supporting the formation of mixed Ti–Si–C phases. These ternary regions are known to enhance interfacial bonding and electrical conductivity.^58^ The oxygen signal in the O map, Figure 4(l), suggests the minimal surface oxidation already observed in Figure 4(e).

**Figure 4:**
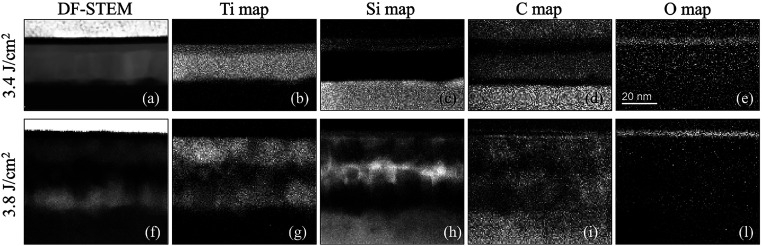
Cross-sectional Dark Field Scanning Transmission Electron Microscopy (DF-STEM) images of 20 nm of Ti deposited on p^+^ 4H-SiC layer irradiated with two distinct laser pulse fluences at (a) 3.4 J/cm^2^ and (f) 3.8 J/cm^2^. The electron energy loss spectroscopy (EELS) elemental maps reveal the distribution of key elements within the irradiated region: Ti (b, g), Si (c, h), C (d, i), and O (e, l), respectively. These maps highlight the changes in elemental composition and diffusion behavior induced by varying laser fluences.

A comparative overview of the structural modifications in Ti/SiC samples following laser irradiation at 3.4 and 3.8 J/cm^2^ is presented in the cross-sectional STEM images shown in Figure 5(a),5(b), respectively. Notably, the overall thickness of the modified region, between the thin top SiO_2_ layer and the underlying SiC anode, differs significantly, measuring approximately 25 nm in Figure 5(a) and 50 nm in Figure 5(b). This difference indicates a more extensive reaction at the higher fluence, reflecting deeper material interaction with the TiC layer entering into direct contact with the SiC. This direct contact, coupled with more advanced interdiffusion and mixing observed in the elemental maps from Figure 4 (bottom row), creates a more continuous, well-bonded interface that supports efficient charge transfer, essential for ohmic behavior. In both cases, a ternary Ti–Si–C alloy phase is present at the top of the modified region, indicating the formation of a mixed intermetallic compound. However, the distribution and bonding of this phase with the surrounding layers vary significantly with the laser fluence, appearing less integrated and more discrete in Figure 5(a), while exhibiting more extensive interdiffusion and better structural integration in Figure 5(b). Overall, these structural differences indicate that increasing the laser fluence significantly enhances the interfacial mixing, phase formation, and crystalline quality of the contact, potentially leading to improved electrical performance. Quantitative analysis of the elemental mapping in Figure 4 indicates that approximately 12 nm of SiC reacted at the lower fluence, while the higher fluence condition resulted in a deeper transformation, with around 30 nm of SiC participating in the reaction. This substantial difference in SiC consumption allows the modified region to extend deeper into the SiC anode, reaching area with higher doping concentrations, and thereby enhancing electrical conductivity. As shown in Figure S1, the simulated aluminum (Al) concentration profile exhibits a slight increase within the first few tens of nanometers, reaching a maximum of 1.2·10^20^ cm^–3^ at a depth of about 150 nm. This initial rise indicates that the Al concentration is not uniform near the surface but increases with depth in the early implanted region. Consequently, the deeper material interaction observed at higher fluence overlaps more effectively with the heavily doped region of the anode. This supports the observed improvement in electrical performance, as it facilitates more efficient carrier injection at the metal–semiconductor interface. A similar effect, where the dopant profile, and thus the local carrier concentration beneath the contact, plays a critical role in contact performance, has been reported for Ni silicide contacts on n-type implanted SiC.^68^ In addition, in Figure 5(a), the structure shows the formation of distinct poorly integrated layers of Si, a predominantly TiC phase that remains physically separated from the underlying SiC. This is consistent with the limited intermixing observed in the elemental maps from Figure 4 (top row), where Ti and Si showed only partial interaction on the top surface. The resulting discontinuous and poorly bonded interface lacks the atomic mixing and electrical continuity required for ohmic behavior. In contrast, in Figure 5(b), a regrown SiC layer is clearly visible, demonstrating substantial interaction and recrystallization at the Ti/SiC interface, along with the formation of a TiC layer, now more continuous and in direct contact with the regrown SiC, covered by a thin Si layer.

**Figure 5:**
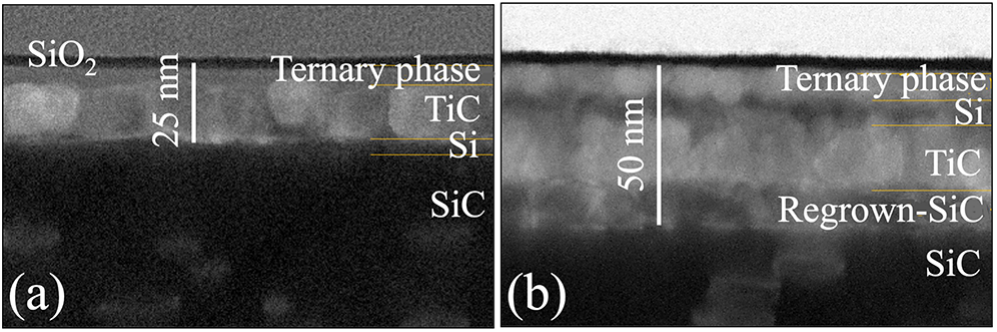
Cross-sectional STEM images of Ti contacts on p+ 4H-SiC layer irradiated with two laser pulses at (a) 3.4 J/cm^2^ and (b) 3.8 J/cm^2^. The images highlight the variation in thickness of the modified region, from the SiO_2_ top layer to the SiC substrate, and the formation of distinct compound layers.

Figure 6 shows the high-resolution TEM images of the sample irradiated with two laser pulses at a fluence of 3.8 J/cm^2^ revealing the formation of well-defined crystalline regions, as confirmed by the corresponding fast Fourier transform patterns. The FFT analysis indicates the coexistence of different crystalline phases within the laser-irradiated region. The presence of a cubic TiC (3C-TiC) structure is identified, suggesting the formation of TiC precipitates resulting from a reaction between titanium and carbon from the underlying SiC matrix. The presence of sharp and symmetric diffraction spots confirms the high crystallinity of these regions. Adjacent to the TiC regions, FFT analysis of the surrounding matrix reveals diffraction patterns corresponding to the cubic polytype of silicon carbide (3C-SiC). These regions exhibit well-defined lattice fringes and an epitaxial relationship with the underlying 4H-SiC epilayer. The simultaneous observation of both 3C-TiC and 3C-SiC nanocrystals, epitaxially grown on the 4H-SiC, suggests that the high-energy laser pulses induced localized melting or amorphization followed by rapid solidification and recrystallization. This phase transformation process enables the nucleation of metastable cubic phases due to their lower interfacial energy and lattice matching with the 4H-SiC. During the investigated PLA processes, the Ti/SiC structure undergoes a transition to a liquid phase, with its local composition varying significantly depending on the laser fluence. The laser fluence controls this liquid phase composition by regulating the energy delivered during the pulse, which in turn influences the extent of melting, atomic diffusion, and interfacial reactions between Ti, Si, and C. The estimated compositions suggest that at the lower fluence of 3.4 J/cm^2^, the liquid phase contains roughly 50% Ti, 25% Si, and 25% C, resulting in a Ti-rich environment that favors the formation of titanium-dominant compounds. In contrast, at the higher fluence of 3.8 J/cm^2^, the relative concentrations shift to approximately 40% Ti, 30% Si, and 30% C, leading to a more balanced composition. This shift supports the formation of Si- and C-rich ternary phases, which are known for their superior interfacial bonding and more homogeneous structure,^58^ both critical for achieving stable ohmic behavior. The transition from a Ti-rich to a more compositionally balanced Ti–Si–C liquid phase at higher fluences fundamentally alters the structural evolution at the interface, promoting the formation of more stable, interconnected phases essential for the ohmic behavior of Figure 3.

**Figure 6:**
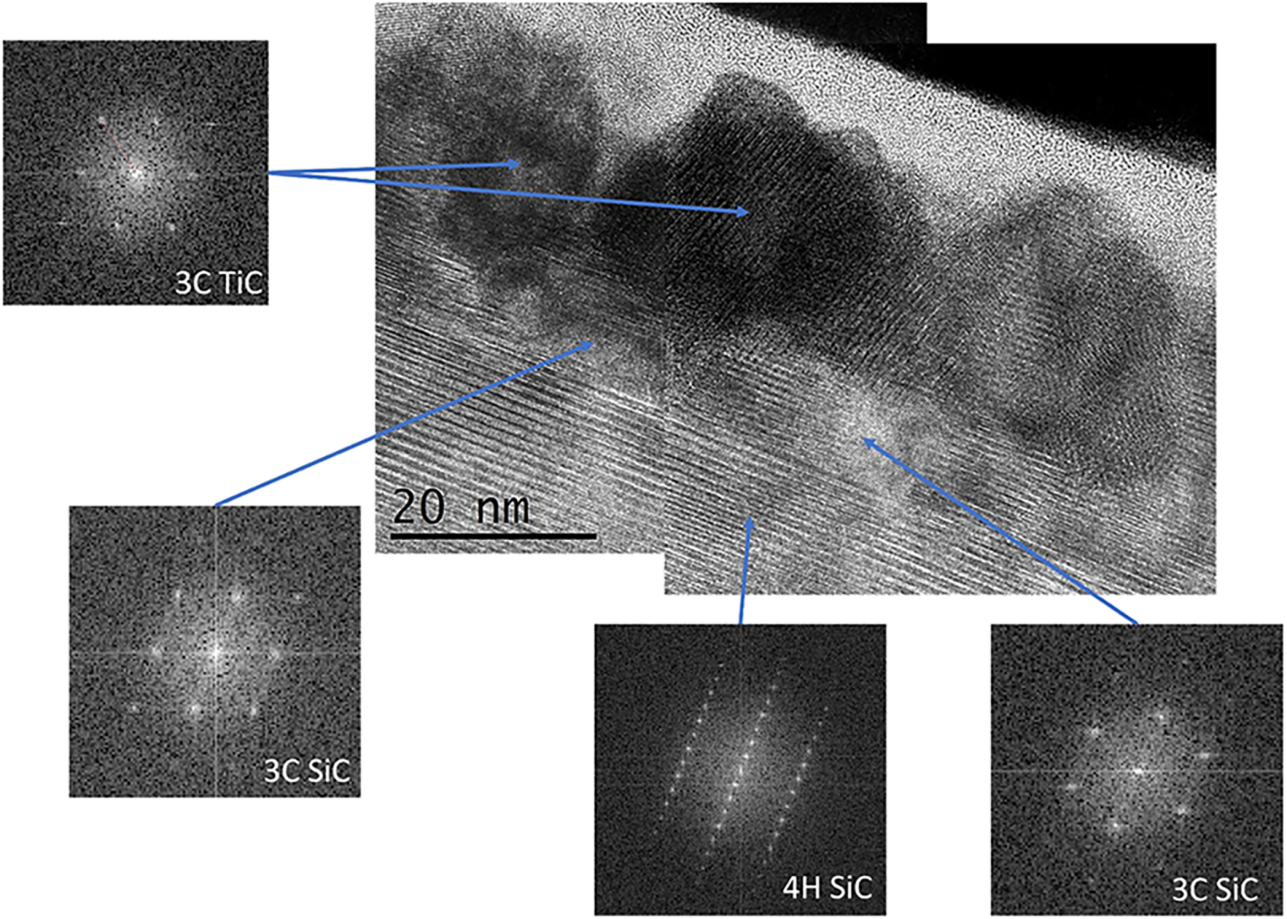
High-resolution TEM images of the sample irradiated with two laser pulses at 3.8 J/cm^2^, with the relative FFT revealing the coexistence of well-defined crystalline regions, including 3C-TiC and 3C-SiC, epitaxially grown on the 4H-SiC epi-layer.

Unlike approaches that rely on specific annealing conditions and Ti/Al ratios to promote Ti_3_SiC_2_ formation, conditions that often include vacuum annealing to prevent Ti oxidation and specific Ti/Al ratios to stabilize this ternary phase,^69^ our results demonstrate that an epitaxially regrown SiC layer bonded to a uniform TiC layer extending into the p-type region underpins the contact performance. PLA’s rapid, localized heating enables these phases without complex multilayers and epitaxial Ti_3_SiC_2_ formation.

## 4 Conclusions

Laser processing, specifically PLA, can effectively tailor the microstructure and electrical properties of Ti metal contacts on p^+^ 4H-SiC regions. The electrical characterization revealed that while 20 nm of Ti contacts annealed under RTA at 1100 °C exhibit nonohmic behavior, by applying PLA with controlled laser fluence, Ti contacts show a marked improvement in ohmic behavior, with linear I–V characteristics emerging above a threshold fluence of 3.6 J/cm^2^. At 3.8 J/cm^2^, conduction improves further, with current levels increasing by more than 50%.

Cross-sectional TEM and elemental mapping provided critical insight into the microstructural changes responsible for the observed electrical improvements. Lower fluence PLA leads to incomplete interfacial reactions and limited mixing between Ti, Si, and C. This results in the formation of a TiC-based alloy layer that remains physically separated from the underlying SiC, impeding electrical ohmic conduction. In contrast, higher fluence PLA promotes a more extensive liquid-phase reaction at the interface, enabling deeper SiC consumption and extension into the anode region. This process facilitates the formation of a continuous, epitaxially regrown SiC layer that is directly bonded to a crystalline TiC layer. The resulting well-integrated interface enhances charge transfer, supporting the formation of a robust ohmic contact. Quantitative analysis indicates that laser fluence not only governs the degree of melting and diffusion but also modulates the liquid-phase composition, from a Ti-rich mixture to a more balanced Ti–Si–C environment. This compositional shift favors the formation of Si- and C-rich ternary phases that exhibit superior interfacial bonding and structural uniformity, which are crucial for achieving stable and low-resistance electrical performance.

Overall, this work demonstrates that PLA enables precise control over interfacial reactions and microstructural evolution. By tuning laser fluence, PLA offers a scalable, selective, and thermally efficient alternative to conventional high-temperature RTA, enabling the formation of well-integrated Ti/SiC interfaces. These results highlight PLA as a promising technique for fabricating Ti ohmic contacts on p-type 4H-SiC, addressing a long-standing challenge in the development of SiC-based power devices.

## Supplementary Material



## Notes

Simulated concentration versus depth profile of Al atoms after implantations and postannealing into SiC (PDF)
