# Supplementary material for: Laser Processing of Ti Contacts for Ohmic Behavior on P‑Type 4H-SiC
Source: ACS Appl Electron Mater. 2025 Sep 16;7(19):9004–11. doi: 10.1021/acsaelm.5c01338 (PMC12530173; doi:10.1021/acsaelm.5c01338)
Supplement: Supplementary file 1 [file el5c01338_si_001.pdf]

Laser Processing of Ti Contacts for Ohmic Behavior on P-Type 4H-SiC

Roberto Vabres[1], Gabriele Bellocchi[2], Corrado Bongiorno[3], Marilena Vivona[3], Fabrizio Roccaforte[3], Paolo Badalà[2], Paola Mancuso[2], Valeria Puglisi[2], Simone Rascunà[2], Isodiana Crupi[1,]*

[1] Engineering Department, University of Palermo, Palermo, 90128, Italy

[2] STMicroelectronics, Catania, 95121, Italy

[3] CNR-IMM, Catania, 95121, Italy

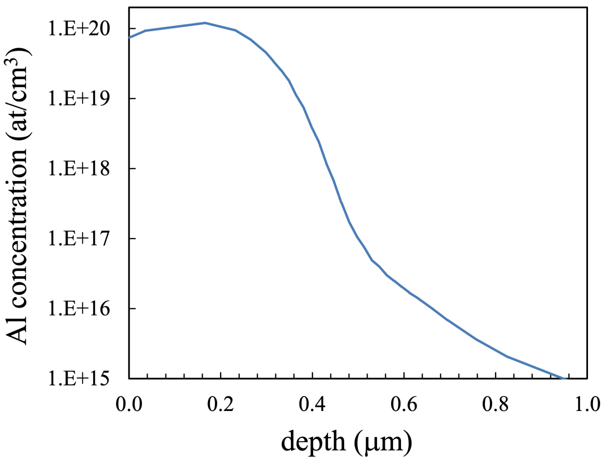

Figure S1. Simulated Al concentration depth profile in SiC after implantation and post-annealing.

* correspondence to Isodiana Crupi, isodiana.crupi@unipa.it
